# Preventing Early Pregnancy and Pregnancy-Related Mortality and Morbidity in Adolescents in Developing Countries: The Place of Interventions in the Prepregnancy Period

**DOI:** 10.1155/2013/257546

**Published:** 2013-01-29

**Authors:** Charlotte Sigurdson Christiansen, Susannah Gibbs, Venkatraman Chandra-Mouli

**Affiliations:** ^1^Department of Maternal, Newborn, Child and Adolescent Health, World Health Organization, Geneva, Switzerland; ^2^Department of Population, Family and Reproductive Health, Johns Hopkins Bloomberg School of Public Health, Baltimore, MD 21205, USA; ^3^Department of Reproductive Health and Research, World Health Organization, Geneva, Switzerland

## Abstract

This paper applies a life-course perspective to the problem of early pregnancy and pregnancy-related mortality and morbidity in adolescents in developing countries. It describes the contribution that two categories of “pregnancy-focused” programmes make—firstly, the provision of effective care and support in the antenatal, childbirth, and postnatal periods (downstream programmes), and secondly, the provision of effective promotive, preventive, and curative care in the prepregnancy period (midstream programmes). It then makes the case for these pregnancy-focused programmes to be set within the context of a third type of programmes, upstream programmes, that is, the provision of promotive and preventive care that contributes to children and adolescents—both male and female—being well nourished, healthy, knowledgeable about their health, and motivated and empowered to protect their health. It provides examples of successful initiatives of all three types of programmes. Finally, it discusses some practical considerations in planning, implementing, and monitoring these three programmes in a coherent manner.

## 1. Introduction

About 16 million adolescent girls between 15 and 19 years old give birth each year, accounting for roughly 11% of all births worldwide. Around 95% of these births occur in developing countries [1]. Evidence suggests that a significant number of girls between 10–14 years old also give birth in some countries. Analysis of surveys conducted in 51 studies from mid-1990s to the early 2000s showed that up to 10% of girls were mothers by the age of 16, with the highest rates in sub-Saharan Africa and South-Central and South-Eastern Asia [2, 3].

For some of these young mothers, pregnancy and childbirth are planned and wanted. For many others they are not. They get pregnant because they are under pressure to marry and to bear children early, because they do not know how to avoid a pregnancy or are unable to obtain condoms and contraceptives to do so, or because they are unable to refuse unwanted sex or to resist coerced sex. Furthermore, when they get pregnant, they are less likely than adults to be able to obtain legal and safe abortions if they want to terminate their pregnancies, or obtain skilled prenatal, childbirth, and postnatal care.

Many adolescents enter pregnancy in poor health and nutritional state [4]. Further, childbirth at an early age is associated with greater health risks for the mother. In developing countries, complications of pregnancy and childbirth are the leading cause of death in young women aged 15–19 years [5]. Because a substantial proportion of adolescent pregnancies are unwanted, many end in abortions—often unsafe abortions. An estimated 3 million unsafe abortions occur globally every year among adolescent girls aged 15–19 years [6].

## 2. The Life-Course Perspective

Interventions that promote the health and wellbeing of children and adolescents can be conceptualized within a life-course framework, a framework which recognizes the importance of early childhood health and development on subsequent outcomes in adolescence and adulthood [7]. In order to prevent early pregnancies and pregnancy-related mortality and morbidity, especially in those adolescents who live in poor families and communities, it is therefore important to provide interventions at various times in the life course. This could be done through:

1. Delivering a package of family planning, safe abortion, and maternal health interventions to adolescents during the antenatal period, childbirth, and the postnatal period.
2. Delivering a package of midstream promotive, preventive, and curative interventions to adolescents in the prepregnancy period.
3. Delivering a package of upstream promotive and preventive interventions to school-age children, both male and female.

## 3. Delivering a Package of Family Planning, Safe Abortion, and Maternal Health Interventions during the Antenatal Period, Childbirth, and the Postnatal Period

Working in collaboration with UNICEF, UNFPA, the World Bank, and the Partnership on Maternal Newborn and Child Health, WHO has developed a package of health interventions to be delivered during a continuum that extends before pregnancy occurs, during pregnancy, childbirth, and after childbirth occurs [8]. The interventions are to be delivered at community level, primary health facility level, and referral level. 

The delivery of family planning can contribute to reducing unintended pregnancies in those adolescents who want to delay or space their pregnancies. Additionally, the delivery of safe abortion and maternal health care during pregnancy, childbirth, and after childbirth occurs can reduce maternal mortality and morbidity in adolescents. 

The delivery of these interventions is a “downstream” response and a partial one. It will not help address the social determinants that contribute to early pregnancy in adolescents, nor will it ensure that adolescents who become pregnant enter pregnancy in good health. This means that they could become pregnant with health problems that have not been detected and treated, for example, anaemia, under/or overweight, or mental disorders (see the Table 1 for listing of health conditions which contribute to maternal mortality and morbidity). 

*Example 1.* The Reproductive Health for Married Adolescent Couples Project (RHMACP) in Nepal included interventions on the individual and community levels to improve the health of married adolescents, by focusing on delaying pregnancy and improving care seeking behaviours before, during, and after pregnancy. Peer educators delivered reproductive health information and distributed condoms and oral contraceptive pills. Workshops and discussion groups were conducted with influential people in the community including community leaders and family members of married adolescents. Project staff worked with local health facilities to enhance youth friendly reproductive health services. An evaluation of the programme showed improvements in sexual and reproductive health knowledge and behaviours among married adolescents, including increased perceived benefits to delaying childbearing. Furthermore, the programme led to increases in knowledge of danger signs during pregnancy, delivery, and the postpartum period. The percentage of married adolescent girls who attended four or more antenatal care visits increased from 29% before the project to 50% after the project. Other encouraging changes included increases in skilled attendance at delivery and use of postnatal care services [9].

## 4. Delivering a Package of Promotive, Preventive, and Curative Interventions in the Prepregnancy Period

Building on reviews done by the Centre for Diseases Control USA, the Agha Khan University Pakistan, the Health Council of the Netherlands, and the Erasmus University Netherlands [10–13], WHO convened a global meeting to develop consensus on a package of interventions to be delivered before pregnancy occurs in order to address health problems, problem behaviours and risk factors that contribute to maternal and childhood mortality and morbidity, and their modes of delivery [14]. Examples of interventions that can be delivered in this period are listed in the Table 1. 

These interventions address areas that are usually part of the maternal and child health agenda, such as nutrition and immunization, and in addition include interventions to address other important areas such as violence, mental health problems, environmental health problems, and tobacco use. The interventions can contribute to reducing unintended pregnancy in adolescents. They can also contribute to reducing mortality and morbidity during pregnancy by ensuring that adolescents enter pregnancy in good health and by increasing the likelihood of adolescents' seeking skilled care during pregnancy and childbirth. They may also contribute to making males aware of the roles they need to play in improving their own health and in contributing to the health of their partners. 

The delivery of these interventions is not quite a downstream response, but neither is it an upstream response. This “midstream” intervention package would add value to the delivery of a package of safe motherhood interventions during the antenatal, childbirth and postnatal periods. But it too would be a partial response because programmes that focus in the immediate prepregnancy period will not be able to undo all the health and nutrition insults suffered in early life. Further, they may not be seen as relevant by girls and young women not contemplating pregnancy, or by boys and young men.

*Example 2.* The Promoting Change in Reproductive Behavior in Bihar (PRACHAR) project in Bihar, India, addressed sexual and reproductive health issues that pertain to both married and unmarried adolescents. Unmarried adolescents aged 15–19 were targeted with a three-day workshop on sexual and reproductive health topics including contraception, nutrition, sexually transmitted infections, and HIV/AIDS. The program successfully delayed marriage of both male and female participants, increased contraceptive use after marriage, and delayed childbearing [15]. PRACHAR reached out to married young people through special events to celebrate newly married couples, which included activities emphasizing the benefits of delaying childbearing and provided couples with a small supply of oral contraceptive pills and condoms [16]. Furthermore, male and female change agents counselled young married men and women individually in their homes on the benefits of delaying childbearing and on other reproductive health topics. The programme led to significant increases in contraceptive demand and contraceptive use among married women under 25 years of age [17].

*Example 3*. The community-based component of the Adolescent Reproductive Health Communication Program (ARH) in Bangladesh included a four-day workshop for adolescents, establishment of adolescent-friendly corners in local health facilities, and distribution of flyers and comic books on adolescent reproductive health topics. These services were targeted at all adolescents aged 10–19 and were intended to increase the use of contraception and condoms among sexually active adolescents, improve adolescent-friendly services, and encourage health seeking behaviours in this target population. The programme led to significant increases in knowledge about pregnancy, maternal health, and contraceptive methods, as well as increased awareness of the drawbacks of early childbearing [18].

## 5. Delivering a Package of Promotive and Preventive Interventions to School-Age Girls and Boys

The FRESH (Focusing Resources on Effective School Health) initiative was launched at the World Education Forum in Dakar in April 2000 by UNESCO, UNICEF, the World Food Programme, WHO, the World Bank, and others [19]. It has four core components that provide the framework for implementing an effective school health and nutrition programme:

1. Policy: health- and nutrition-related school policies that are nondiscriminatory, protective, inclusive, and gender-sensitive.
2. School environment: safe and psychologically supportive, with access to safe water and separate sanitation facilities for girls, boys, and teachers.
3. Education: skills-based education that addresses health, nutrition, HIV prevention, and hygiene issues and promotes positive behaviours.
4. Services: simple, safe, and familiar health and nutrition services that can be delivered cost-effectively in schools and increase access to youth-friendly clinics [19].

The delivery of these components together can contribute to ensuring that older children (5–9 years) and adolescents are healthy and well informed about health and illness, and about what they need to do to stay well and to get back to good health when they are ill. Even as importantly, it can help build attitudes and values that are positive and respectful of oneself and others, which may contribute to healthy and prosocial behaviours later in life.

Improving the nutritional status and health of adolescents, as well as stimulating and supporting them to take charge of their lives, can lead to multiple health and social benefits then and later on, including preventing early and unintended pregnancies and poor pregnancy-related outcomes. 

*Example 4*. The Tawana Pakistan Project provided school lunches at girls' primary schools in selected rural districts of the country in order to increase school attendance of girls and improve their nutritional status. Local women were selected and trained to plan and prepare balanced meals to more than 418,000 students over the course of the project. The program significantly reduced wasting and underweight and the enrolment of participating schools increased by 40% during the intervention [20, 21].

*Example 5*. The Saloni Swasth Kishori Yojana programme targets adolescent girls in Uttar Pradesh, India. Services provided include iron and folic acid (IFA) supplementation, biannual deworming, medical examination, and education on topics including nutrition, hygiene, the legal age of marriage, and the benefits of delaying childbearing [22, 23]. The programme is targeted to in-school girls aged 10–19 years [22]. A pilot study of the project with 11–14-year olds in-school girls showed that the intervention increased uptake of IFA supplementation and health checkups [23].

## 6. Discussion

Many adolescents die or experience lasting complications during childbirth. Many do so because they do not get the care and support they need during childbirth. Ensuring that adolescents obtain skilled and sensitive childbirth and postpartum care will reduce these deaths.

Many adolescents enter pregnancy in a poor nutritional state; experiencing health problems including communicable diseases such as sexually transmitted infections and malaria and noncommunicable diseases such as depression; practicing unhealthy behaviours such as tobacco use and alcohol abuse and living in risky physical environments (e.g., spending hours in environments filled with smoke from wood-burning cooking stoves) and risky social environments (e.g., living with an abusive partner). Many do not get the promotive, preventive, and curative health and social services they need. Ensuring that adolescents make the WHO-recommended four clinic visits during the antenatal period will increase the likelihood of healthy pregnancies and healthy outcomes for mothers and babies. Extending the provision of promotive, preventive, and curative care to the prepregnancy period will further increase the likelihood of these outcomes. In addition, they will reduce some unintended pregnancies. 

Even in the poorest of countries, strengthened child survival programmes are helping reduce the number of child deaths from vaccine-preventable diseases, pneumonia, diarrhoea, malaria, and HIV. There are, however, hardly any programmes for older children and younger adolescents. This group needs a range of interventions including access to preventive and curative health services. Older children and adolescents need to be educated, vaccinated, administered nutritional supplements, screened for health problems, and treated when ill. These are not pregnancy-related interventions but could make enormous contributions to reducing early and unintended pregnancies, and complications during pregnancy and childbirth.

These efforts could be led and managed by a mix of national public health programmes that focus exclusively on adolescents and by other programmes that address adolescents as an integral part of the population. Whatever administrative and institutional arrangements are used, there should be specific commitments on adolescent health in national health policy and strategy documents. There should also be specific attention to adolescents within programmatic actions—for example, epidemiology, advocacy, health service provision, and community action. 

## 7. Conclusion

Using a life-course framework to prevent early pregnancy and reduce pregnancy-related morbidity and mortality in adolescents provides the basis for adding two layers of intervention packages to the traditional maternal health package of antenatal care, delivery care, and postnatal care. The first layer is a package of promotive, preventive, and curative interventions in the prepregnancy period, and the second is a package of promotive and preventive interventions to school-age girls and boys even before reproductive capacity has developed.

Adolescence has been described as a time of opportunity and of risk. The layered approach described in this paper will contribute to minimising this risk and maximising the opportunity that this special life phase brings [24].

## Figures and Tables

**Table 1:** See [14].

	Illustrated examples of preconception care interventions
Nutritional conditions	Supply iron and folic acid Screen for anaemia Monitor nutritional status Provide nutrition education, and counselling

Vaccine-preventable diseases	Vaccinate against rubella, tetanus, and hepatitis B

Genetic conditions	Provide genetic screening, counselling and testing where needed Discuss reproductive outcomes and options and provide the appropriate services

Environmental health	Provide guidance on how to avoid unnecessary exposure to radiation, pesticides and lead Promote the use of improved stoves and cleaner liquid/gaseous fuels

Infertility/subfertility	Increase awareness and understanding on infertility and sub-fertility Screen, diagnose and treat for infertility and sub-fertility

Female Genital Mutilation (FGM)	Increase awareness and understanding on female genital mutilation Screen to detect complications and provide information on where to get treatment Carry out defibulation of infibulated or sealed girls and women Treat complications

Too-early, unwanted, and rapid successive pregnancies	Educate girls and boys about sexuality Provide contraceptive information and services Link health service provision to activities aimed at: (i) reducing marriage before the age of 18 years; (ii) keeping girls in school

Sexually transmitted infections	Provide age-appropriate sexual health education and safer sex promotion Promote condoms Screen and treat for sexually transmitted infections

HIV	Provide age-appropriate sexual health education and safer sex promotion Provide HIV counselling and testing Provide treatment for HIV-related illnesses when needed Enrol women in mother to-child transmission (PMTCT) programmes, antenatal care programmes and provide family planning services

Interpersonal violence	Recognize signs of violence against women Provide medical and psychosocial care Link health service provision to activities aimed at: (i) changing harmful gender norms; (ii) bringing about gender equality; (iii) empowering girls and women economically

Mental health	Provide information and counselling Diagnose and manage mental health and neurological conditions Link health service provision to activities aimed at strengthening community networks and women's empowerment

Psychoactive substance abuse	Screen for and—where needed—treat substance abuse Provide information and counselling to reduce substance use

Tobacco use	Screen for tobacco use (smoking and smokeless tobacco) Provide information and counselling on tobacco cessation Provide information on the dangers of second-hand smoke
